# Insights Into the Emergence of Paroxysmal Nocturnal Hemoglobinuria

**DOI:** 10.3389/fimmu.2021.830172

**Published:** 2022-01-28

**Authors:** Melissa A. Colden, Sushant Kumar, Bolormaa Munkhbileg, Daria V. Babushok

**Affiliations:** ^1^ Division of Hematology-Oncology, Department of Medicine, University of Pennsylvania, Philadelphia, PA, United States; ^2^ Comprehensive Bone Marrow Failure Center, Department of Pediatrics, Children’s Hospital of Philadelphia, Philadelphia, PA, United States

**Keywords:** paroxysmal nocturnal hemoglobinuria, PIGA, GPI-anchored, aplastic anemia (AA), bone marrow failure (BMF), autoimmunity, complement, clonal hematopoeisis

## Abstract

Paroxysmal Nocturnal Hemoglobinuria (PNH) is a disease as simple as it is complex. PNH patients develop somatic loss-of-function mutations in phosphatidylinositol *N*-acetylglucosaminyltransferase subunit A gene (*PIGA*), required for the biosynthesis of glycosylphosphatidylinositol (GPI) anchors. Ubiquitous in eukaryotes, GPI anchors are a group of conserved glycolipid molecules responsible for attaching nearly 150 distinct proteins to the surface of cell membranes. The loss of two GPI-anchored surface proteins, CD55 and CD59, from red blood cells causes unregulated complement activation and hemolysis in classical PNH disease. In PNH patients, *PIGA*-mutant, GPI (-) hematopoietic cells clonally expand to make up a large portion of patients’ blood production, yet mechanisms leading to clonal expansion of GPI (-) cells remain enigmatic. Historical models of PNH in mice and the more recent PNH model in rhesus macaques showed that GPI (-) cells reconstitute near-normal hematopoiesis but have no intrinsic growth advantage and do not clonally expand over time. Landmark studies identified several potential mechanisms which can promote PNH clonal expansion. However, to what extent these contribute to PNH cell selection in patients continues to be a matter of active debate. Recent advancements in disease models and immunologic technologies, together with the growing understanding of autoimmune marrow failure, offer new opportunities to evaluate the mechanisms of clonal expansion in PNH. Here, we critically review published data on PNH cell biology and clonal expansion and highlight limitations and opportunities to further our understanding of the emergence of PNH clones.

## Introduction

**Figure 1:**
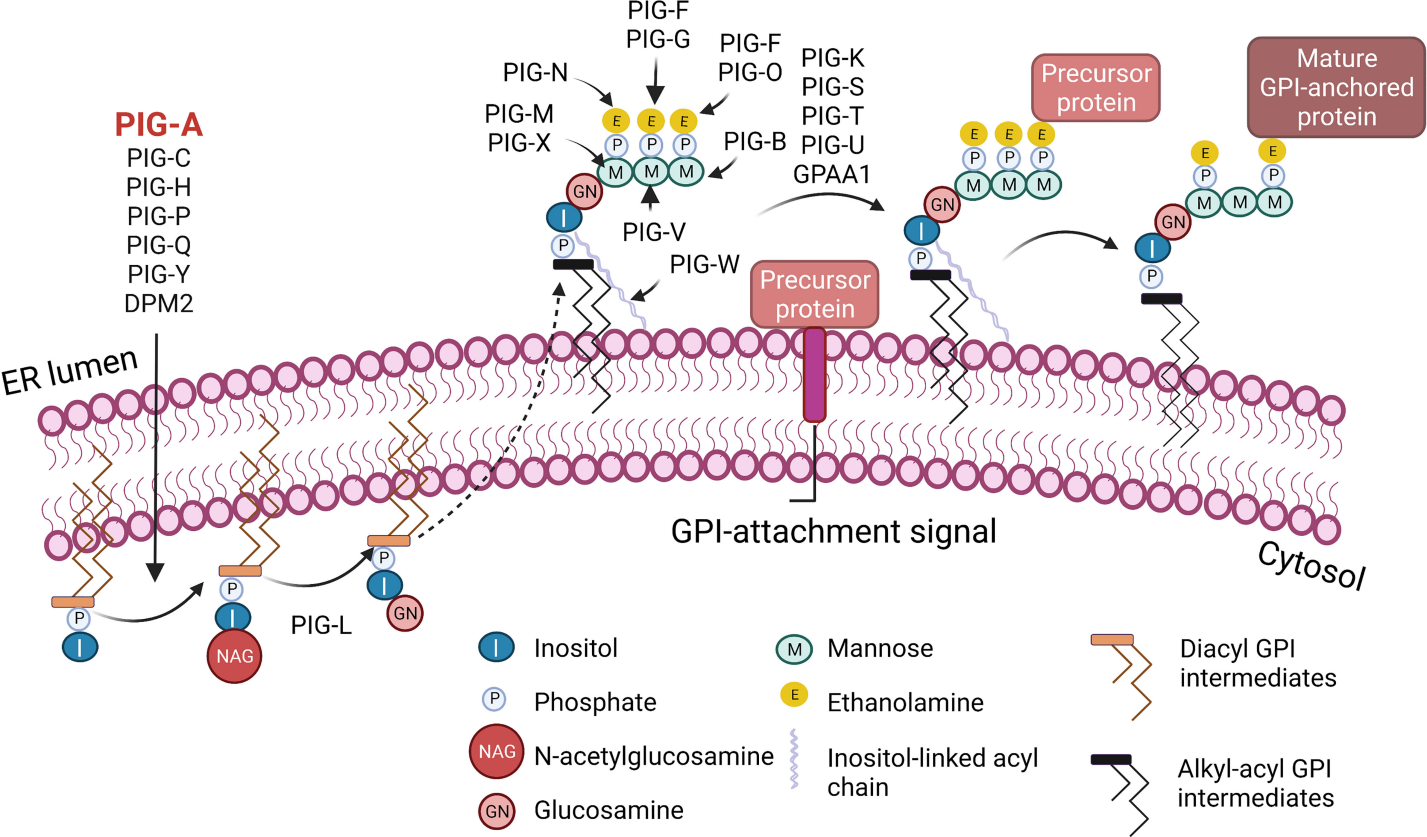
Biosynthesis of glycosylphosphatidylinositol (GPI)-anchored proteins. GPI-anchored proteins are surface proteins linked to the membrane through a GPI glycolipid attachment. GPI is synthesized through a series of steps on the membrane of the endoplasmic reticulum (ER). The initial step involves the transfer of N-acetylglucosamine to inositol on the cytoplasmic side of the ER membrane by a multi-subunit enzyme comprised of seven proteins that include *PIGA*. A series of subsequent steps, most of which occur on the luminal side of the ER membrane, produce the mature GPI moiety, which is then transferred to the C-terminus of the precursor protein that contains a GPI-anchor attachment signal. Following attachment of GPI, the GPI can be further modified and remodeled during its transport through the Golgi network on the way to the cell surface. I, Inositol; M, Mannose; P, Phosphate; E, Ethanolamine; NAG, N-acetylglucosamine; GN, Glucosamine. Figure created with BioRender.com.

**Figure 2:**
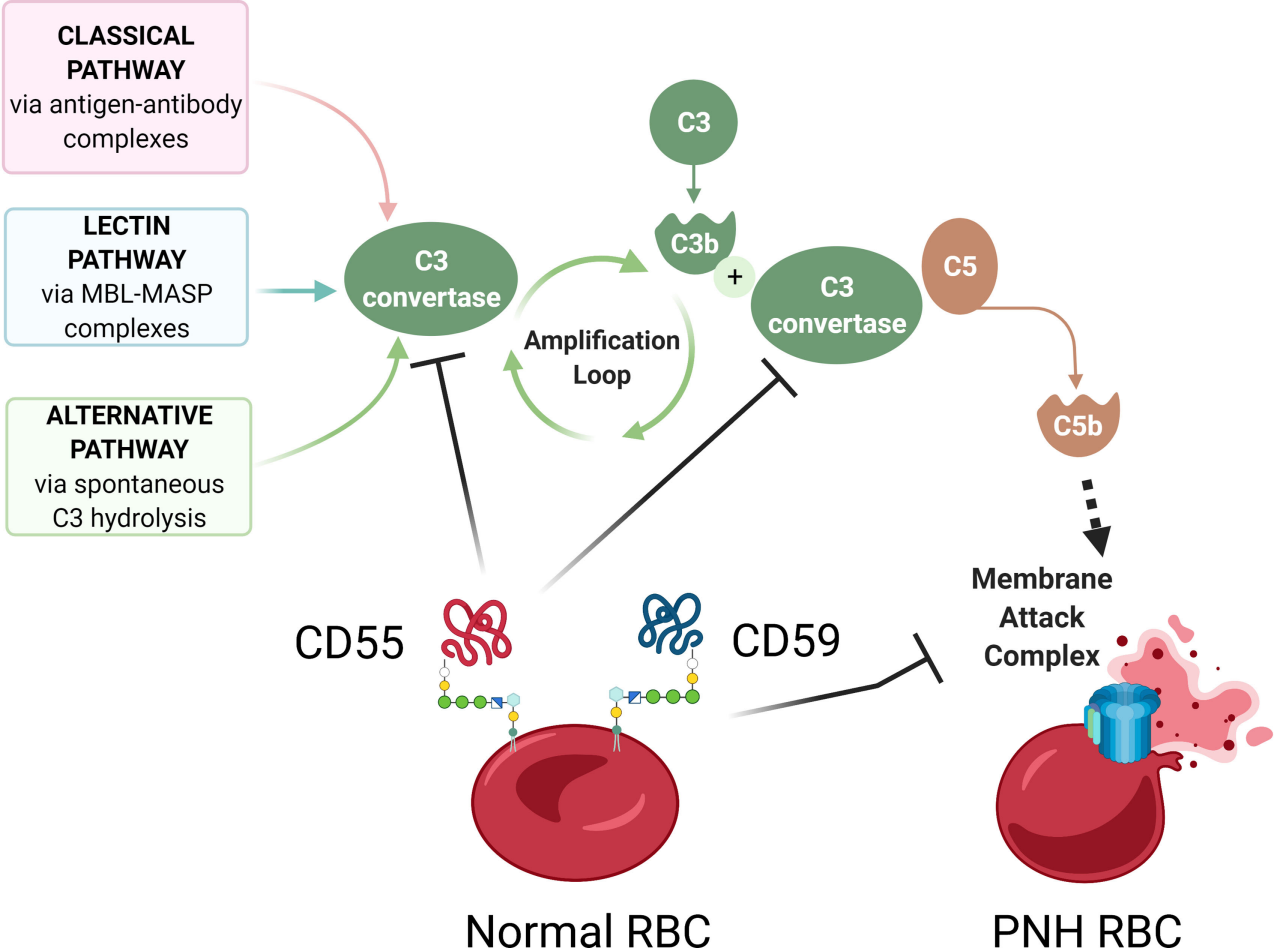
Complement-mediated hemolysis in PNH. A schematic diagram showing aberrant complement activation on PNH erythrocytes due to the deficiency of GPI-anchored proteins CD55 and CD59. The Classical, Lectin, and Alternative pathways of complement activation lead to the formation of C3 convertase, which cleaves C3 into C3a and C3b, leading to formation of C5 convertase, which cleaves C5 to C5b, to activate terminal complement components and form the membrane attack complex (MAC). In normal red blood cells (RBCs), GPI-anchored surface proteins CD55 and CD59 inhibit complement activation by blocking C3 convertase and membrane attack complex, respectively. In the absence of CD55 and CD59, GPI-deficient PNH RBCs have uncontrolled complement activation and lysis. Diagram made with BioRender.com; complement pathways adapted from “Roles of the Complement Cascade in Innate Immunity” by BioRender.com (2021).

Paroxysmal Nocturnal Hemoglobinuria (PNH) is a rare, life-threatening blood disease characterized by hemolysis, propensity for blood clotting, and bone marrow failure (1). Although PNH was recognized in the clinic as early as 1882 by Dr. Paul Strübing, it was over a century later when the cause of PNH was identified as an absence of a group of membrane proteins attached to the cell surface by glycosylphosphatidylinositol (GPI) anchors (1–3). In most PNH patients, GPI-anchor deficiency is caused by somatic mutations in phosphatidylinositol glycan anchor biosynthesis class A (*PIGA*), an X-linked gene required for GPI anchor biosynthesis (4–6) (**Figure 1**). *PIGA* mutations occur in early hematopoiesis, and subsequent clonal expansion of *PIGA*-mutant cells lacking GPI-anchored proteins (GPI-APs) leads to clinical PNH disease. In this review, we will refer to *PIGA*-mutant cells as GPI (-) or PNH cells. Loss of GPI-APs is particularly detrimental for human erythrocytes, which in the absence of complement regulatory proteins CD55 and CD59 become susceptible to uncontrolled complement activation, leading to hemolytic anemia (**Figure 2**).

**Figure 3:**
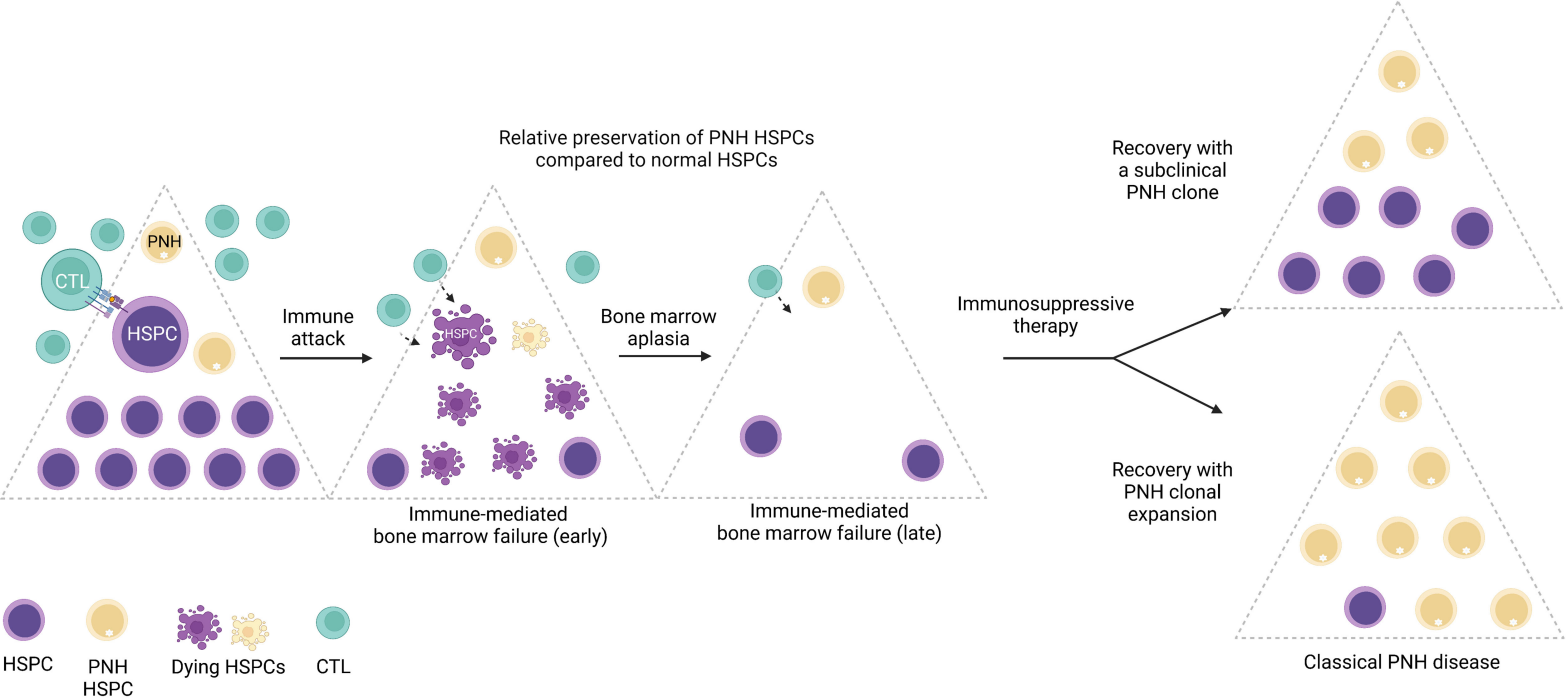
The emergence of PNH clones is closely related to immune-mediated bone marrow failure. A schematic diagram illustrating the relationship between immune-mediated bone marrow failure (acquired aplastic anemia) and clonal expansion of HSPCs with somatic mutations in the *PIGA* gene (PNH HSPCs). In acquired aplastic anemia, cytotoxic T lymphocytes (CTL) recognize an unknown autoantigen presented by the hematopoietic stem and progenitor cells (HSPCs). Aberrant recognition of the HSPCs leads to their autoimmune destruction (shown as dying HSPCs) and manifests clinically as bone marrow failure. PNH HSPCs are hypothesized to have relative resistance to autoimmune destruction in AA. In AA patients treated with immunosuppressive therapy, bone marrow may recover with a smaller subclinical PNH clone; however, in a subset of patients, PNH HSPCs undergo progressive clonal expansion, leading to classical PNH disease, characterized by hemolytic anemia and related symptoms, and an increased risk of thrombosis. Figure created with BioRender.com.

While large clonal expansions of GPI (-) cells cause hemolytic PNH disease, tiny stable populations (~0.001% to 0.005%, 1 to 5 per 10 million cells) can be found in most healthy individuals (7, 8). In contrast, more expanded PNH clones develop in nearly 50% of patients with immune-mediated acquired aplastic anemia (AA), an autoimmune bone marrow aplasia caused by autoreactive T lymphocyte attack on hematopoietic stem and progenitor cells (HSPCs) (9–11). The close relationship between PNH and AA was first noted over 60 years ago by Dr. William Dameshek in his famous editorial (12), but the nature of the link between the two conditions continues to be a subject of intense speculation (1, 13–17). The frequent co-occurrence of PNH with immune-mediated AA and lack of several immunologically relevant GPI-anchored surface molecules on PNH HSPCs have led to the prevailing theory that PNH HSPCs may have a relative survival advantage during autoimmune attacks in AA patients, leading to clonal expansion of PNH HSPCs upon recovery in a subset of patients treated with immunosuppression (17, 18) (**Figure 3**).

In this review, we critically examine existing animal models of PNH, comparing experimental observations with human PNH disease and discussing the challenges of cross-species modeling of PNH. We will highlight seminal results arising from animal models and studies of patient cells and discuss remaining controversies and opportunities to expand our understanding of the emergence of PNH disease and its relationship to AA.

## Development of Mouse Models of *PIGA* Loss and GPI-Anchored Protein Deficiency

In 1993, somatic mutations in *PIGA* gene were identified as the cause of PNH (4–6, 19–21). Although over 20 distinct enzymes are required to produce mature GPI anchors (**Figure 1**), PIGA is the only gene in this pathway located on the X chromosome. A single inactivating *PIGA* mutation is sufficient to disrupt GPI biosynthesis and eliminate all GPI-APs from *PIGA*-mutant cells, both in males and females (due to X chromosome inactivation). Currently, there are 139 human proteins known to be GPI-anchored, and several more are predicted to be GPI-anchored based on computational and proteomic analyses (22–24). GPI-APs participate in various cellular processes, including immune response regulation, signal transduction, and cell-cell adhesion (25, 26). Multiple rare congenital syndromes of GPI-anchored protein deficiencies have been described, one of which is multiple congenital anomalies-hypotonia-seizures syndrome 2 (MCAHS2) (27). MCAHS2 is a rare X-linked disorder caused by germline hypomorphic mutations in *PIGA*, leading to early childhood lethality from severe anomalies that include structural malformations of the central nervous system (27–30). In contrast to the inherited *PIGA* mutations in MCAHS2, *PIGA* mutations in PNH are acquired somatically and are restricted to hematopoietic cells, which remain viable and can reconstitute trilineage hematopoiesis (31).

**Figure 4:**
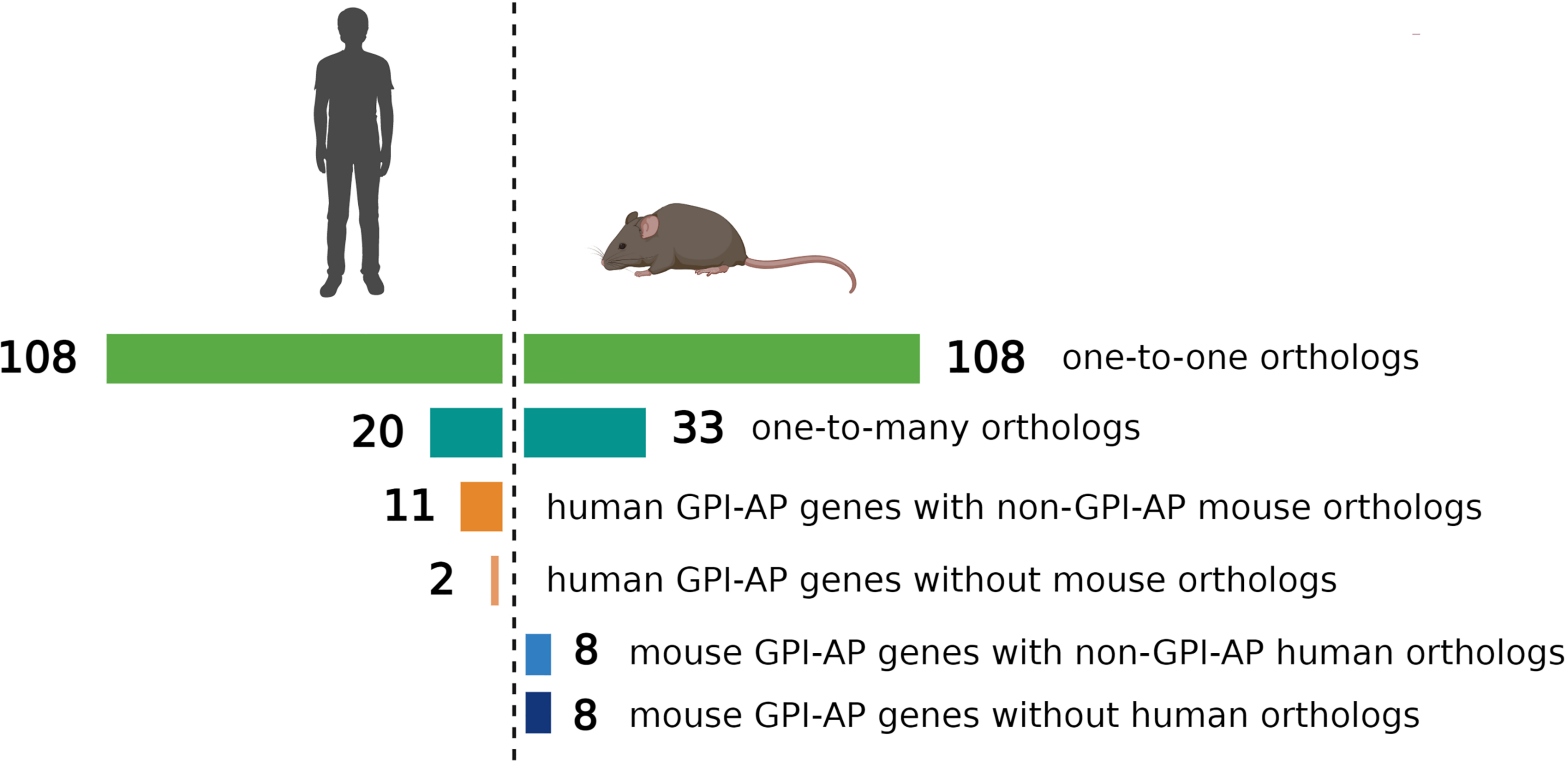
Comparison of evolutionary conservation of GPI-anchored proteins between humans and mice. 108 human GPI-AP genes had one-to-one orthologs in mouse, 20 human GPI-AP genes had more than one mouse ortholog (n=33), 11 human GPI-AP genes were orthologous to mouse genes whose products were not GPI-anchored, and 2 human GPI-AP genes lacked mouse orthologs. 8 mouse GPI-AP genes were orthologous to human genes whose products were not GPI-anchored, and 8 mouse GPI-AP genes lacked human orthologs. Figure adapted from Kumar and Babushok (24).

**Figure 5:**
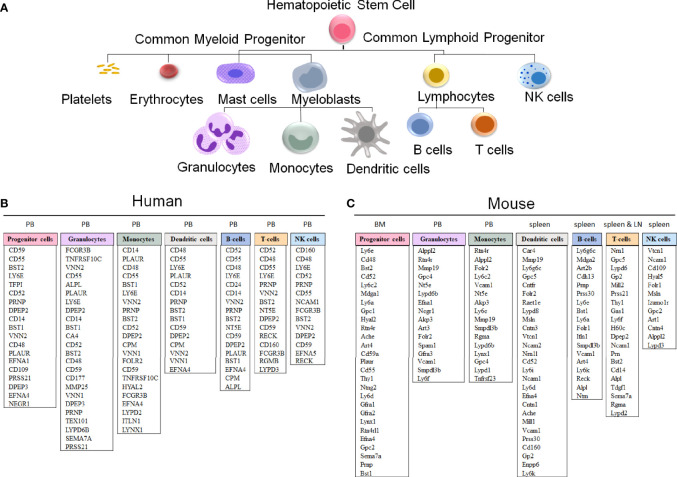
GPI-anchored protein genes in human and mouse hematopoietic cells. **(A)** A schematic representation of hematopoietic cell differentiation, with the corresponding lists of GPI-AP genes with the highest expression in sorted hematopoietic cell populations in humans **(B)** and mice **(C)**. The subsets of the more highly expressed GPI-AP genes, defined as having ≥ 10 transcripts per million (tpm) in published RNA-sequencing datasets, are listed in the descending order of expression within each of the cell lineages. **(B)** The human GPI-AP gene expression are listed based on RNA-Seq from specified peripheral blood (PB) subsets by Monaco et al., 2019 (48), available at the Human Blood Atlas. **(C)** The mouse GPI-AP genes expressed at ≥ 10 tpm are shown based on RNA sequencing expression analysis of mouse (C57BL/6) hematopoietic cell subsets isolated from Bone Marrow (BM, progenitor cells; Bolden et al, 2018 (49)), PB (granulocytes and monocytes), spleen (dendritic cells and T cells published by Marsman et al, 2018 (50); B-cells published by Shi et al, 2015 (51)) and lymph nodes (LN) (T cells published by Marsman et al, 2018 (50))), as extracted from the Haemosphere data portal (52, 53).

The evolutionary conservation of *PIGA* and the synteny of the mammalian X-chromosome led to the development of *Piga*-mutant mice to model PNH (**Table 1**). However, in cross-species modeling of *PIGA* deficiency, differences in GPI-anchoring status and functional divergence of various GPI-APs present a unique challenge. In contrast to mouse models of other single-gene defects, a knockout of *Piga* results in loss of not just *Piga* but all GPI-anchored proteins from the surface of mutant cells. A recent comparative analysis of GPI-AP conservation between humans and mice showed that the majority of GPI-APs are conserved, with 77% having one-to-one mouse GPI-AP orthologs and >90% of human GPI-AP genes having at least one GPI-anchored mouse ortholog (24). However, there were also several differences, with 13 human genes having no GPI-anchored orthologs in mice and, conversely, 16 mouse GPI-AP genes not having corresponding GPI-anchored orthologs in humans (**Figure 4**) (24). While divergence of GPI-AP genes with no expression in hematopoietic cells is less consequential to PNH modeling, an evolutionary divergence of those expressed in hematopoietic lineages (**Figure 5**) can lead to unanticipated phenotypic differences between PNH mice and PNH patients. Of practical relevance, the absence of GPI-APs has complicated the analysis of the HSPC compartment in mice because of their lack of classical GPI-anchored surface markers Sca1 and CD48 used for immunophenotypic analyses of murine HSPCs.

**Table 1:** Summary of animal models of paroxysmal nocturnal hemoglobinuria.

Reference	Model	Tissue-specificity/Cre	Selected Observations
**Germline *Piga* loss mouse model**			
Kawagoe et al. (1996) (32)	Constitutional *Piga* knockout; mice chimeric for *Piga* deficiency.	n/a	Low chimerism in non-hematopoietic tissues due to embryonic lethality in mice with high proportion of GPI (-) cells. Percentage of GPI (-) hematopoietic cells did not increase over time.
Rosti et al. (1997) (31)	Constitutional *Piga* knockout; mice chimeric for *Piga* deficiency	n/a	In mice chimeric for *Piga* deficiency, *Piga* embryonic stem cells were competent for trilineage hematopoiesis; there was no expansion of GPI (-) cells.
**Chimeric mouse model**			
Tremml et al. (1999) (33) Keller et al. (1999) (34)	Constitutional *Piga* knockout; mice chimeric for *Piga* deficiency	EIIa-Cre; early embryonic	Mosaic mice are viable. GPI (-) RBCs have increased sensitivity to complement mediated lysis and shorter half-life; comparatively high fraction of GPI (-) lymphocytes; no clonal expansion of GPI (-) cells overtime.
**Hematopoietic-specific *PIGA* loss mouse model**			
Keller et al. (2001) (35)	Hematopoietic-cell-restricted knockout of *Piga*	Fes-Cre; all hematopoietic cells	GPI (-) cells can fully reconstitute trilineage hematopoiesis, have differences in lineage contribution (e.g., more GPI (-) T cells), but have no clonal expansion overtime.
Jasinski et al. (2001) (36)	Erythroid/megakaryocyte- restricted knockout of *Piga*	GATA1-Cre; erythroid-megakaryocyte lineage	Leaky expression in early embryogenesis leading to high embryonal lethality. Mice that escaped embryonal recombination had almost 100% of red cells with partial deficiency of GPI-anchored proteins, and intermediate sensitivity to complement, resembling type II PNH cells.
Visconte et al. (2010) (37)	Hematopoietic-cell-restricted knockout of *Piga*	Fes-Cre; all hematopoietic cells	No hemolysis; the frequency of GPI (-) cells was much higher in T lymphocytes but lower in erythrocytes, granulocytes, and B cells.
Hazenbos et al. (2004) (38)	T lymphocyte-restricted knockout of *Piga*	Lck-Cre; T lymphocytes	Stimulation by ConA or Allogeneic stimulation of GPI (-) T cells induced higher proliferative responses than normal cells.
Hazenbos et al. (2011) (39)	Hematopoietic-cell-restricted knockout of *Piga*	Vav-Cre; all hematopoietic cells	Grossly normal numbers of T and B lymphocytes, monocytes, neutrophils, and erythrocytes; detailed hematopoietic analysis not performed.
**Fetal liver transplant mouse model**			
Murakami et al. (1999) (40) Murakami et al. (2002) (41)	A transplant model of hematopoietic cell-restricted knockout of *Piga*	CMV-Cre, followed by transplantation of fetal liver cells from female mice mosaic for *Piga*-deficiency into wild type recipients.	No expansion of GPI (-) cells overtime. Frequency of GPI (-) cells was highest in T lymphocytes and immature thymocytes. GPI (-)cells engrafted following transplantation, but did not outcompete wild type cells. When transplanted with CD4^+^ allo-reactive to donor hematopoietic cells, GPI (-) donor cells were less sensitive to CD4^+^ T cell-driven immune attack.
**GPI-anchored complement regulator knockout mouse model**			
Sun et al. (1999) (42)	GPI-DAF-Deficient Mice	n/a	No embryonic lethality; no hemolytic anemia
Lin et al. (2001) (43)	*Daf*-1 knockout mice	excision in ESCs	No hemolytic anemia; increased C3 deposition on erythrocytes of *Daf-1* ^-/-^ mice.
Holt et al. (2001) (44)	*Cd59a* knockout mice	n/a	No hemolytic anemia; elevated reticulocytes; erythrocytes susceptible to complement lysis.
Miwa et al. (2002) (45)	*Cd59a* knockout mice and *Cd59a*/*Daf double* knockout mice	n/a	High sensitivity to complement lysis but no spontaneous hemolytic anemia.
Qin et al. (2003) (46)	Cd59b knockout mice	n/a	Spontaneous hemolytic anemia with morphological abnormalities in RBCs and platelets.
**Non-human primate model**			
Shin et al. (2019) (47)	CRISPR/Cas9 *PIGA* gene editing in Rhesus Macaque	n/a	No clonal expansion of GPI (-) cells.

n/a, not applicable.

### Constitutional *Piga* Deletion in Mice Causes Embryonic Lethality Due to Multiple Extrahematopoietic Abnormalities

The early development of *Piga*-mutant mice was complicated by embryonic lethality. When a vector targeting *Piga* gene was transfected into murine embryonic stem cells (ESCs) followed by their injection into blastocysts to create chimeric mice (31, 32), litter sizes were smaller than expected. Surviving mice had very low (1-5%) chimerism with no germline transmission of *Piga*-null allele, suggesting that GPI-APs are essential for embryonic development (31). *Piga-*mutant ESCs also failed to produce normal embryoid body (EB) structures *in vitro*, with resultant EBs structurally disorganized and smaller than expected (31, 54).

Embryonic lethality was subsequently confirmed in the EIIa*-Cre*/*Piga-LoxP* model which deleted *Piga* in early embryogenesis under the control of the adenovirus EIIa promoter (34). EIIa-*Cre* is active in oocytes and preimplantation embryos, therefore female embryos with various degrees of mosaicism for *Piga-*mutant cells were produced. Heterozygous female mice with high levels of Cre recombination did not survive after birth due to multiple abnormalities, including orofacial malformations that caused feeding difficulties. X-inactivation analysis of mice with high Cre recombination rates revealed significantly skewed silencing of the X-chromosome carrying the null *Piga* allele in kidney, heart, lung, brain, and liver, suggesting that GPI (-) cells in these tissues die or are at a growth disadvantage. Partially recombined mice who survived to adulthood had complete Cre recombination within oocytes but produced no offspring bearing a *Piga*-null allele, consistent with embryonic lethality.

Subsequent studies using conditional *Piga* inactivation with keratin 5-Cre (K5Cre), expressed in basal cells of the skin, revealed lethality within a few days of birth due to abnormal skin development (55), reinforcing the importance of GPI-APs in nonhematopoietic tissues. The extrahematopoietic manifestations of *Piga* deletion explain the early childhood lethality seen with hypomorphic germline PIGA mutations in patients with MCAHS2 syndrome. However, hematopoietic tissues of EIIa-*Cre*/*Piga-LoxP* mice had nearly equal proportions of recombined and wild-type cells, consistent with no detrimental effect of *Piga* loss for hematopoietic development (34).

### *Piga*-mutant Hematopoietic Cells Reconstitute Trilineage Hematopoiesis but Have No Cell-Intrinsic Growth Advantage

To model *Piga* loss in hematopoietic cells, several mouse models of hematopoietic cell-specific *Piga* deficiency have been developed (31, 33, 34, 36, 37, 40, 54, 56) (**Table 1**); of which two landmark studies were most informative in evaluating the role of GPI-AP deficiency in hematopoiesis (34, 40).

Murakami et al. created a transplant-based model of *Piga* deletion. In this model, *Piga-LoxP*/Y males were first mated with females bearing a broadly expressed human cytomegalovirus-Cre (hCMV-Cre) to produce heterozygous embryos mosaic for GPI-anchor deficiency due to X-inactivation (40, 57). Fetal liver cells from mosaic day 14 embryos were then transplanted into irradiated wild-type mice. The recipient mice were evaluated for reconstitution by donor GPI (+) and GPI (-) cells, and the relative contribution of GPI (-) cells to various peripheral blood cell lineages was tracked for evidence of clonal expansion over time (40).

In a separate model, Keller et al. produced a hematopoietic-specific knockout of *Piga* using a Cre/LoxP system under the control of the *c-fes* promoter region, which turns on in embryonic development and is active in definitive hematopoiesis (35). In this model, *Piga* excision was incomplete and occurred gradually, requiring up to eight months to achieve complete recombination in male *fes-Cre* homozygous, *Piga-LoxP* mice. Aged mice in this model had neuromuscular symptoms, attributed to the “leakiness” of the Cre expression, with recombination detected in some neural cells.

Both models demonstrated stable proportions of GPI (-) blood cells over the animals’ lifetime without clonal expansion in up to 42 weeks of follow-up. GPI (-) cells reconstituted near-normal hematopoiesis, with no or only minimal differences in blood counts between mutants and wild-type mice (35, 40). Transplant studies showed no apparent defects in repopulating capacity in primary and secondary transplantation assays (35, 40).

More recently, advances in gene editing allowed the creation of the first non-human primate model of PNH (47). Shin et al. used CRISPR/Cas9 gene editing to disrupt *PIGA* in CD34^+^ HSPCs in rhesus macaques. *PIGA*-mutant CD34^+^ HSPCs were then autologously transplanted into two animals, with the resultant GPI (-) cell proportions tracked by flow cytometry over time. Similar to the stability of PNH cell populations in mice, the GPI (-) cell populations persisted at a low, stable frequency of 0.2% to 0.6% for more than two years of follow-up without clonal expansion, confirming the absence of cell-intrinsic growth advantage of PNH cells.

### Altered Lymphopoiesis in Mice Mosaic for *Piga* Deficiency

While the early mouse models of PNH were mired by difficulties in achieving complete *Piga* loss in hematopoietic cells, the ability to track GPI (-) cell populations by flow cytometry allowed studies of competitive advantage in animals mosaic for *Piga* loss. One crucial advantage of analyzing mosaic animals is the ability to track the relative contributions of GPI (-) and GPI (+) cells within various cell lineages, whereby GPI (+) cells serve as the “built-in” wild type control akin to a competitive situation in human patients with somatic *PIGA* mutations.

Immunophenotypic analysis of GPI (-) cell proportions in *Piga* mosaic mice unexpectedly revealed significant alterations in GPI (-) lymphopoiesis. T cell populations in peripheral blood of *Piga* mosaic animals in all PNH mouse models were comprised predominantly of GPI (-) cells, with the reverse skewing to GPI (+) cells in the B cell compartment (33, 34, 37, 40). Evaluation of GPI (-) and (+) cell proportions in immature thymocytes showed that the skewing to GPI (-) T cells occurred early in thymocyte development (40). The reasons for the disproportionate contribution of GPI (-) cells to T lymphopoiesis have not been experimentally defined but have been hypothesized to involve a possible inhibitory role of GPI-APs in thymic homing or T lineage commitment. Notably, a skewing to GPI (-) cells is not observed in the T cell lineage in PNH patients (**Table 2**), pointing to possible differences in GPI-AP involvement in human versus mouse lymphopoiesis. Alternative explanations for the smaller fractions of GPI (-) T lymphocytes in patients could involve factors such as the longevity of lymphocytes, acquisition of *PIGA* mutations later in life, and varying selection pressures on GPI (-) cells of lymphoid and myeloid lineages.

**Table 2:** Comparison of hematologic findings in PNH patients and mouse models of PNH.

Findings	Hematopoietic mosaic mice generated by fetal liver cell transplant. Murakami et al. (1999) (40)	Hematopoietic mosaic LF female mice at 16 mos of age. Keller et al. (2001) (35)	Hematopoietic mosaic mice generated by fetal liver cell transplant with alloreactive CD4^+^ T cells. Murakami et al. (2002) (41)	Bone marrow failure with associated PNH clone (58, 59)	Classical PNH patients (58, 59)
Clonal expansion	No	No	Yes	Yes; mean granulocyte clone 11% (58, 59)	Yes, mean granulocyte clone >70% (58, 59)
Immune pressure	No	No	Yes	Yes	Yes
Hemolytic anemia	No	No	No	Varies	Yes
GPI (-) Myeloid cell	Stable ~20-30% engraftment	~50% GPI (-): GPI (+); no clonal expansion	Transient GPI (-) advantage post-BMT		Favors GPI (-)
GPI (-) T lymphocyte	Favors GPI (-)	Favors GPI (-)	Transient GPI (-) advantage post-BMT		Favors GPI (+)
GPI (-) B lymphocyte	Stable ~20-30% engraftment	<50% GPI (-); no clonal expansion	Transient GPI (-) advantage post-BMT		Favors GPI (+)
GPI (-) Red blood cells	Stable ~20-30% engraftment	~50% GPI (-): GPI(+) reticulocytes; no clonal expansion	ND		Favors GPI (-)

ND, not determined.

Interestingly, non-mosaic mice with complete *Piga* deletion in T lymphocytes in the *Lck-Cre*/*Piga-LoxP* model showed no gross aberrations in T cell subsets (38), suggesting that the competitive environment in *Piga* mosaic mice likely exposes the differences between GPI (-) and GPI (+) lymphocytes. Functionally, GPI (-) T cells are competent in response to protein antigens, but have increased proliferation in response to lectin-based stimulant Concanavalin A (Con A) (38). Visconte et al. also reported abnormalities in T regulatory cell development, with smaller proportions of GPI (-) CD4^+^CD25^+^FoxP3^+^ T regulatory cells in mosaic mice (37).

### GPI (-) Mouse Erythrocytes Have Mild Sensitivity to Complement Without Overt Hemolytic ANEMIA

While PNH patients suffer from severe hemolytic anemia, *Piga*-mutant mice have normal or near-normal red blood cell counts (33, 35, 40). Careful examination of hematologic parameters and complement sensitivity identified a shorter half-life and a heightened sensitivity to complement-mediated lysis of GPI (-) erythrocytes with compensatory reticulocytosis. However, these abnormalities were much milder than in human patients and did not result in hemolytic anemia (**Table 2**). An even milder phenotype was found in an erythroid-specific knockout of *Piga* driven by a GATA1-Cre. *GATA1-Cre*/*Piga-LoxP* knockout mice had only a partial GPI-AP deficiency, similar to type II erythrocytes in humans. The intermediate levels of GPI-APs were explained by GATA1-Cre turning on later in erythroid development, leading to the persistence of residual GPI-APs in mature erythrocytes (36).

The lack of intravascular hemolysis in *Piga-*mutant mice was the most notable cross-species difference in modeling PNH. Unlike humans, mice have a unique transmembrane complement regulatory molecule Crry, which inhibits C3 convertase at the cell surface, functioning similar to the human GPI-AP decay-accelerating factor (DAF, CD55) (60–62). Additionally, unlike the single GPI-anchored isoform for human DAF, mice also have a non-GPI-anchored DAF isoform. These cross-species differences in complement regulation explain why GPI (-) erythrocytes in mice are only mildly sensitive to complement. As a comparison, a combined knockout of Crry and CD59 led to 10 to 15 times increased erythrocyte complement sensitivity compared to control mice, and ~3-5 times higher complement sensitivity than *Piga-*mutant mice (40, 63). Notably, while these differences limit the utility of *Piga*-mutant mice for studies of complement-mediated hemolysis, the lack of hemolytic anemia and normal viability of PNH mice has been advantageous for long-term studies of PNH clonal expansion and HSPC biology.

## The Search for External Factors Responsible for Clonal Expansion of PNH Cells

*Piga*-mutant cells’ lack of intrinsic growth advantage in animal models provides strong evidence that *PIGA* mutations alone are insufficient to cause PNH disease and that additional external factors are necessary for GPI (-) cell clonal expansion. Several possible hypotheses have been proposed; the most rigorously investigated were the reduced sensitivity to apoptosis and inflammatory cytokines by PNH cells and PNH cell resistance to various immune-mediated attacks.

### GPI (-) and GPI (+) Cells in PNH Patients Have Different Responses to Apoptotic Stimuli

Resistance to apoptotic stimuli has been one of the most hotly debated mechanisms of PNH clonal expansion (64–76) (**Table 3**). Brodsky et al. reported improved preferential survival of GPI (-) cells (granulocytes, CD34^+^ progenitors, and lymphoblastoid B-cell lines) compared to GPI (+) cells under conditions of serum starvation. GPI (-) lymphoblastoid cells were also more resistant to radiation injury than cells corrected for *PIGA* (68). Follow-up studies using an inducible TF-1 erythroleukemia cell line model of *PIGA* loss revealed lower apoptosis rates of GPI (-) cells in multiple cell killing assays (using allogeneic mononuclear cells or NK92 natural killer cell as cytotoxic effectors, or inducing apoptosis by high dose TNF-α or γ-irradiation) (76). Other groups reported similar results, with reduced apoptosis rates and lower Fas antigen expression in GPI (-) cells (e.g., granulocytes or progenitors) compared to GPI (+) cells in PNH patients (67, 72, 75). However, subsequent observations challenged the conclusion that GPI (-) cells are intrinsically resistant to apoptosis.

**Table 3:** Summary of studies evaluating response to apoptosis by PNH cells.

Manuscript	Model	Condition Tested	Main Findings
Brodsky et al., 1997 (68)	Comparison of CD59^+^ and CD59- granulocytes from PNH patients (n=4), progenitors (n=2), and B lymphoblastoid cell lines (n=2).	Survival of cells in serum-free media over 24-72 hours (all cells), resistance to radiation (B cell lines).	GPI (-) cells had improved survival.
Horikawa et al., 1997 (65)	Unsorted peripheral blood granulocytes and lymphocytes from patients with PNH (n=11), AA (n=13), MDS (n=12) and healthy controls (n=20). CD34^+^ cells from PNH patients (n=8).	Induction of apoptosis using anti-Fas antibody in granulocytes and lymphocytes. Induction of apoptosis in CD34^+^ cells with 4 day pretreatment with IFN- γ, TNF-α, followed by anti-Fas antibody.	Unsorted granulocytes from patients (PNH, AA, and MDS) were resistant to apoptosis; there was no correlation between apoptosis resistance and clone size (including clones 10%). BM CD34^+^ cells from PNH patients (and MDS patients) had increased resistance to apoptosis compared to controls.
Ware et al., 1998 (70)	Granulocytes from PNH patients (n=26) and healthy controls (n=20); GPI (-) lymphoblastoid B cell line and cell line corrected for *PIGA*.	Rate of apoptosis after serum starvation (granulocytes), and serum starvation, γ-radiation and anti-Fas antibody (cell lines).	Apoptosis rate in response to serum starvation was lower in granulocytes of PNH patients compared to controls. There were no differences in Fas antigen expression. Apoptosis rate did not correlate with PNH clone size. There were no differences in apoptosis of GPI (-) and GPI (+) B lymphoblastoid lines after serum starvation, γ-radiation, and anti-Fas antibody treatment.
Chen R et al., 2000 (73)	Comparison of CD59^+^ and CD59- CD34^+^ progenitors from PNH patients and healthy controls in liquid culture.	Cell growth and differentiation capacity.	GPI (+) cells from PNH patients had worse growth compared to GPI (-) cells and compared to CD34^+^ cells from controls, accompanied by higher expression of CD95 (Fas receptor) and higher sensitivity to Fas antibody treatment.
Chen G et al., 2002 (72)	Comparison of GPI (-) and GPI (+) CD34^+^ progenitors from PNH patients and healthy controls.	Growth in liquid culture and methylcellulose assays, apoptotic markers.	GPI (-) cells produced more progenitors and outcompeted GPI (+) cells in mixing experiments; however, this was not due to increased proliferation. Instead, GPI (+) cells had more apoptotic cells and higher Fas expression. After removing apoptotic cells, the growth of GPI (+) and GPI (-) cells was similar.
Kulkarni et al., 2002 (71)	Comparison of apoptosis sensitivity of GPI (+) and GPI (-) cells (thymocytes, granulocytes, and hematopoietic progenitor cells in Fes-Cre and EIIa Cre Piga-LoxP mouse model of PNH	Apoptosis in response to exposures to γ-irradiation, dexamethasone, etoposide, and anti-Fas antibody, and whole body γ-irradiation of mice.	No differences in apoptosis rates in GPI (-) and GPI (+) cells in response to various apoptotic stimuli.
Ismail et al., 2003 (67)	CD34^+^ cells from PNH patients (n=10) and healthy controls (n=18)	Assessment of viability, Fas expression in GPI (+) and GPI (-) CD34^+^ cells from the same patients	The viability of CD34^+^ cells in PNH patients was lower than in healthy controls, which was predominantly due to the GPI (+) cell subset. GPI (+) cells had higher expression of Fas antigen than GPI (-) cells.
Yamamoto et al., 2002 (77)	Unsorted granulocytes from PNH patients (n=5) compared to granulocytes from healthy volunteers (n=5).	The proportion of apoptotic cells, Fas antigen expression, and caspase-3 activity in unsorted granulocytes of PNH patients versus controls.	No differences in rates of apoptosis, Fas antigen expression, or Caspase-3 activity in PNH vs. control granulocytes.
Chen et al., 2005 (74)	Gene expression analysis of pooled GPI (-) and GPI (+) fractions of CD34^+^ progenitors from PNH patients, and CD34^+^ patients from controls	Gene expression	Gene expression of GPI (-) progenitors from PNH patients was similar to progenitors in controls, while GPI (+) progenitors from patients had upregulation of apoptotic proteins and other changes.
Savage et al., 2008 (66)	CD34^+^ progenitors from PNH patients (n=6) and healthy controls. Inducible *PIGA*-mutated TF1 cell line.	Assessment of apoptosis after induction with TNF-alpha, γ-radiation. Evaluation of cytotoxicity from allogeneic PBMCs and NK92 cell line.	CD34^+^ GPI (-) cells were more resistant to autologous cell-mediated killing, as well as allogeneic cell-mediated killing. Lower cytotoxicity from allogeneic PBMCs or NK92 cells in GPI (-) TF1 cells, compared to GPI(+) TF1 cells. Lower apoptosis induction of GPI (-) TF1 in response to TNFa or gamma-irradiation.
Kunyaboon et al., 2012 (75)	Comparison of CD59^+^ and CD59- granulocytes from PNH patient (n=15), and controls (n=33).	Apoptotic rate after 0-4 hours culture in the presence and absence of mononuclear cells or autologous CD8^+^ cells.	GPI (+) granulocytes had a higher proportion of apoptotic cells than GPI (-) granulocytes at 0 and 4 hours of culture. Co-culture with mononuclear cells increased the differential between apoptotic fractions in GPI (+) and GPI (-) cells. Percent apoptotic GPI(+) cells in PNH patients was higher than in healthy controls.

The first clue came from studies showing that while unfractionated granulocytes of PNH patients were more resistant to apoptosis, the apoptotic rates did not correlate with the patients’ PNH clone sizes (65, 70). Subsequent comparisons of apoptotic rates in cells from PNH patients and healthy controls showed that, unexpectedly, the differences in apoptosis between GPI (-) and GPI (+) cells were caused by higher cell death in “normal” GPI (+) cells of PNH patients (72–74). The dying GPI (+) cells were already present at the time of initial blood collection, and their removal eliminated all subsequent differences in apoptotic susceptibility and Fas antigen expression; thus, suggesting that the *in vivo* environment in patients led to GPI (+) cell death (72). Mouse models of PNH confirmed no intrinsic differences in apoptotic rates in *Piga*-mutant cells. Kulkarni et al. found no differences in cell death between GPI (-) and GPI (+) hematopoietic cells (thymocytes, granulocytes, and bone marrow hematopoietic progenitors from *Fes-Cre/Piga-LoxP* mice) exposed to γ-irradiation, etoposide, dexamethasone, anti-Fas antibody, or when mice were treated with whole-body γ-irradiation (71).

Together, these data suggest that GPI (+) and GPI (-) cells may have distinct responses to certain physiologic stimuli *in vivo*, leading to higher GPI (+) cell death and increased relative survival of GPI (-) cells in PNH patients. The inciting triggers have not been defined but could involve altered responsiveness to inflammatory cytokines, hematopoietic growth factors, or HSPC-directed autoimmune cytotoxicity, among other possibilities. Other factors not captured in animal or cell culture models of PNH are the cumulative differences between GPI (+) and GPI (-) cells, which may be present in patients due to varied clonal evolution, replicative history, telomere attrition, and cell senescence. Additionally, comparisons of apoptotic rates in GPI (+) and GPI (-) cells could be confounded by the known effects of GPI-APs on the externalization of phosphatidylserine unrelated to apoptosis, which could cause a relative increase in Annexin V staining in non-dying GPI (+) cells compared to GPI (-) cells (78).

### Inflammatory Cytokine Responses by GPI (-) and GPI (+) Cells

The data on the effects of inflammatory cytokines on clonal expansion in PNH are conflicting but suggest that GPI (-) cells may respond differently to inflammatory cytokines under certain conditions *in vitro* (65, 66, 79, 80). For example, the addition of IFN-γ and TNF-α to methylcellulose media selectively reduced the fraction of GPI (+), favoring the growth of GPI (-) colonies in colony-forming assays using bone marrow mononuclear cells from PNH patients (79). Similarly, GPI (-) erythroleukemia TF1 cells had higher viability than GPI (+) TF1 cells when cultured with high doses of TNF-α (66).

In contrast to these *in vitro* studies of patient cells or TF1 cell lines, extensive studies of cytokine sensitivity in PNH mice showed no intrinsic differences in cytokine responses between GPI (-) and GPI (+) murine HSPCs. When treated with IFN-γ, TNF-α, MIP-1α, or TGF-β_1_, GPI (-) HSPCs from *Fes*-*Cre*/*Piga*-*LoxP* mice had no advantage over GPI (+) control cells in hematopoietic progenitor assays and did not outcompete GPI (+) cells in bone marrow transplants (80). Similarly, *in vivo* treatment with polyinosinic-polycytidylic acid (Poly I:C), which induces many proinflammatory cytokines, did not increase the proportion of GPI (-) cells in animals mosaic for *Piga* mutation (80).

Taken together, the available data suggest that there could be conditions under which patients’ PNH cells may respond differently to inflammatory cytokines. However, the molecular mechanisms underlying these differences and whether altered cytokine responsiveness in cultured cell assays is determined solely by *PIGA* loss or whether it depends partly on additional, unaccounted factors in GPI (+) versus GPI (-) patient cells are unknown. The degree to which cytokine-mediated selection contributes to PNH pathogenesis under physiologic conditions *in vivo* also remains unclear.

### Mechanisms of Immune Escape by PNH Cells

**Figure 6:**
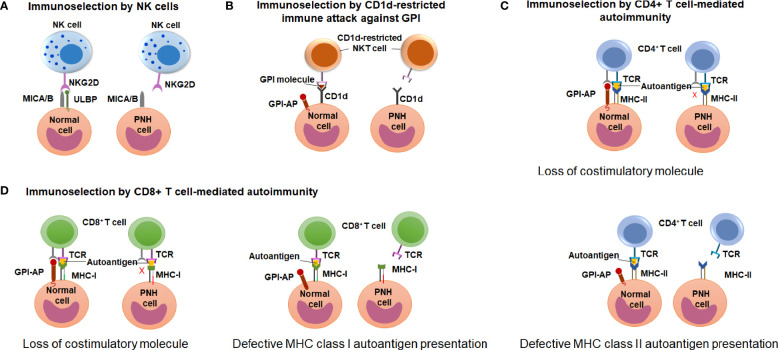
Potential mechanisms of immune-escape by the PNH cells. **(A)** A schematic diagram illustrating the hypothesis of immunoselection of GPI (-) cells by NK cells. Normal GPI (+) cells express NKG2D ligands ULBPs and MICA/B, and activate NK cells (left), whereas the lack of ULBPs on GPI **(-)** PNH cells leads to an impaired NK activation (right). **(B)** A schematic diagram illustrating the immunoselection of GPI (-) cells by CD1d-restricted immune attack against GPI. CD1d-restricted, GPI-specific NKT cells target GPI molecule presented by CD1d and attack GPI (+) normal HSPCs (left) but not the GPI (-) PNH HSPC (right). **(C)** A schematic diagram illustrating the hypothesis of immune selection of GPI (-) cells by CD4^+^ lymphocyte-mediated attack. GPI (-) cell may have the reduced ability to activate autoreactive CD4^+^ T lymphocytes due to missing GPI-anchored co-stimulatory molecules (top diagram). Alternatively, GPI (-) PNH cells may have reduced MHC class II presentation of hypothetical autoantigens compared to GPI (+) normal cells, and survive CD4^+^ T cell mediated immune attack due to their reduced recognition (bottom diagram). **(D)** A schematic diagram illustrating the hypothesis of immune selection of GPI (-) cells by CD8^+^ lymphocyte-mediated attack. GPI (-) cell may have the reduced ability to activate autoreactive CD4^+^ T lymphocytes due to missing GPI-anchored co-stimulatory molecules (left diagram). Alternatively, GPI (-) PNH cells may have reduced MHC class I presentation of hypothetical autoantigens compared to GPI (+) normal cells, and survive CD8^+^ T cell mediated immune attack due to their reduced recognition (right diagram).

Early observations of the unique association between PNH and immune-mediated AA have led to wide speculation that clonal expansion of GPI (-) cells occurs because GPI (-) cells escape HSPC-directed immune attack in AA (12, 17, 18). However, the search for immune escape mechanisms has been hindered by an incomplete understanding of the immune pathogenesis of AA. Candidate approaches led to several proof-of-concept demonstrations of immunologic scenarios where GPI (-) cells may have an upper edge, showing immunoselection of GPI (-) cells by 1) natural killer (NK) cell-mediated cytotoxicity (81–83); 2) NKT cell-mediated attack targeting GPI bound to CD1d molecules (84, 85); and 3) CD4^+^ T cell-mediated alloimmunity (41) (**Figure 6**). How these contribute to GPI (-) cell selection in AA and PNH patients is still a matter of active debate.

#### Immunoselection of GPI (-) Cells by NK Cells

Nagakura et al. used *PIGA*-mutant and *PIGA*-corrected leukemia cell lines to test the sensitivity of GPI (-) cells to NK-mediated immune attack (81). When co-cultured with NK cells from healthy volunteers, *PIGA*-mutant cell lines were killed at lower rates than control cells. NK cytotoxicity does not involve Fas ligand but instead is dependent on perforin; accordingly, blocking perforin in these assays abrogated NK-mediated cytotoxicity. However, when purified perforin was used instead of NK cells, *PIGA*-mutant and control cell lines were killed at similar rates, indicating that differences in NK-mediated killing likely involved NK cell activation (81). The reasons for improved survival of *PIGA*-mutant lines were subsequently narrowed down to the lack of GPI-anchored stress-inducible surface molecules, ULBP1, ULBP2, and ULBP3 (83). ULBP proteins serve as ligands for the natural-killer group 2, member D (NKG2D) receptors on NK, natural killer T (NKT), and some conventional CD8^+^ T cells. Blocking ULBPs with monoclonal antibodies reduced NKG2D activation and NK-mediated cell killing in co-culture assays (83).

It has been more difficult to establish the role of ULBPs in autoimmune HSPC destruction and PNH clonal expansion in AA and PNH patients. Nearly 50% of PNH patients and 60% of AA patients have detectable ULBP 1, 2, or 3 on GPI (+) granulocytes, and all evaluated AA and PNH patients had detectable ULBPs on CD34^+^ hematopoietic progenitors (82, 83). However, ULBPs were almost always co-expressed with non-GPI-anchored stress-inducible NKG2D ligands MICA and MICB (82, 83). Cell killing assays using autologous NK cells and NKG2D-expressing T cells demonstrated killing of granulocytes from PNH patients expressing NKG2D ligands (83), and blocking NKG2D by antibodies improved hematopoietic progenitor colony formation by bone marrow mononuclear cells from AA and PNH patients (82). However, ULBP ligand dependence of NKG2D-mediated cytotoxicity could not be confirmed because patients cells also expressed MICA/B ligands. Moreover, in contrast to the preferential killing of GPI (+) cells in the K562 and two lymphoid leukemia cell line models of PNH, the same differential sensitivity of patients’ GPI (+) cells compared to GPI (-) cells was not demonstrated (82, 83). Discrepant results between NK-mediated killing of cell lines compared to patients’ cells could be at least partially explained by HLA class I expression differences. K562 and MOLT-4 cell lines, used in NK cytotoxicity studies, lack HLA class I expression and are thus more susceptible to NK-mediated killing (86–88). In contrast, increased inflammatory cytokines (e.g., interferon-γ) in AA patients upregulate HLA class I expression, inhibiting NK cell activation *in vivo.* Furthermore, detection of NKG2D ligands on mature granulocytes of AA patients (which are not targeted by autoimmune attack in AA) and the presence of ULBP3 on granulocytes in healthy controls demonstrate that NKG2D ligand expression alone is insufficient to induce NK-mediated cell killing *in vivo*. Indeed, from an evolutionary standpoint, unregulated NK cell activation by binding to NKG2D ligands on stressed hematopoietic cells would be highly detrimental.

In considering the role of NKG2D-mediated cytotoxicity in AA and PNH, additional questions remain. While NKG2D ligands aside from ULBP3 were not detected in healthy controls and disappeared in AA patients in remission, over a third of patients with myelodysplastic syndrome (MDS) had detectable NKG2D ligands. Although NKG2D ligand expression in patients experiencing inflammatory stress or patients with inherited marrow failure syndromes was not explored, the detection of NKG2D ligands in MDS raises a question of a more general NKG2D ligand induction in stress hematopoiesis. Broad activation of NKG2D ligands in stress hematopoiesis would not account for the unique association of PNH with AA, as PNH clonal expansion is not seen in other marrow failure diseases (89, 90). Additionally, NKG2D-mediated killing in AA and PNH does not account for the clinical efficacy of T cell-mediated therapies and the recently demonstrated MHC class I-restriction of autoimmune attack in AA (91–94).

#### Immunoselection of GPI (-) Cells by CD1d-Restricted Immune Attack Against GPI

Gargiulo et al. proposed that the target of autoimmunity in PNH and AA is the glycolipid molecule GPI itself, and that the absence of GPI in *Piga*-mutant cells allows them to escape GPI-directed autoimmune attack (85, 91, 92). Unlike peptide antigens presented by HLA molecules and recognized by conventional T cells, lipids are presented by monomorphic HLA class I-like molecules CD1d and recognized by a specialized family of lymphoid cells, the natural killer T cells (NKT cells) (93). GPI was previously identified as a natural ligand for CD1d (94), although its specific function as a CD1d ligand is still debated (94–97).

Gargiulo et al. found a small population of PNH patients’ CD8^+^ lymphocytes that could bind to CD1d dimers loaded with GPI molecules and demonstrated that this lymphocyte population was several-fold larger in PNH patients compared to controls (85). CD8^+^ T lymphocytes from PNH patients but not healthy controls were activated by co-culturing them with GPI-loaded CD1d-expressing leukemic cell lines (which lacked HLA class I expression). Lymphocyte activation was highest when both GPI and CD1d were present, suggesting that lymphocytes likely recognized GPI bound to CD1d (85). A parallel study of AA patients identified a similarly expanded CD1d-restricted T cell population in AA (84). However, not all AA patients had CD1d-restricted T cells, raising doubts about their role in AA pathogenesis (84). Moreover, the presence and relative amounts of CD1d-restricted T cells did not correlate with the presence and size of PNH clones, as would have been expected if CD1d-mediated autoimmunity was responsible for PNH immune selection (84).

Although Gargiulo et al.’s results demonstrated that PNH and AA patients have expanded populations of CD8^+^ lymphocytes capable of being activated by CD1d-GPI complexes *in vitro*, questions as to the identity of GPI/CD1d-reactive lymphocytes and their role in the pathogenesis of AA and PNH remain. While published studies did not distinguish NKT cells from conventional CD8^+^ T lymphocytes, the CD1d-restriction of the identified cell populations suggests that these cells are likely CD8^+^ NKT cells. Because the identified cells lacked the invariant Vα24-Jα18 T cell receptor (TCR) rearrangement that characterizes type I NKT cells in humans, the identified cells were likely type II NKT cells, which have a more varied TCR repertoire (93).

NKT cells arise during thymic development through a specialized differentiation process and are functionally distinct from conventional T cells. When a double-positive (DP) thymocyte produces a T cell receptor (TCR) rearrangement that can bind to CD1d on neighboring DP thymocytes, the CD1d-responsive thymocyte is selected to differentiate down the NKT cell lineage (98). Unlike HLA, CD1d lacks a binding site for CD4 or CD8 co-receptors, and most mature NKT cells are CD4^-^ CD8^-^ double-negative (DN) or CD4^+^; however, rare CD8^+^ NKT cells can occur (98). Because Gargiulo et al. focused their analysis on CD8^+^ cells, it is not known whether the identified CD1d-restricted CD8^+^ cells represent a fraction of the larger population of GPI/CD1d-reactive NKTs in AA and PNH patients or if CD8^+^ NKT cells are uniquely expanded. Notably, in an independent study, Mannick et al. had previously evaluated the frequency of CD3^+^ CD1d-tetramer^+^ NKT cells in two PNH patients (97). In agreement with Gargiulo’s findings, Mannick et al. also found an expanded population of NKT cells (0.28% and 0.095% of peripheral blood mononuclear cells) compared to 0.029% in 22 controls (97).

Importantly, the available data are insufficient to establish the role of CD1d-restricted GPI-reactive cells in HSPC-directed cytotoxicity in AA and the significance of expanded GPI-reactive NKT populations in AA and PNH pathogenesis remains uncertain. The proposed model of GPI-directed immune attack needs to account for the tissue specificity of autoimmunity in AA, despite the ubiquitous presence of GPI and the broad expression of CD1d on non-hematopoietic tissues (99). CD1d-restriction of NKT cell-mediated attack also contradicts the recent genetic evidence of MHC class I-restriction of autoimmune attack in AA (100, 101). An alternative explanation for increased CD1d-restricted NKT cells in AA and PNH is that they may have an immunomodulatory function. Type II NKT cells can modulate the activation of conventional T cells, type I NKT cells, B cells, and granulocytes and even affect the integrity of epithelial cells, causing suppression or activation of immune responses depending on context (93). In several models of autoimmunity, e.g., a model of experimental autoimmune encephalomyelitis (102), type II NKT cells have an immunomodulatory function and alleviate autoimmunity. Thus, additional studies are needed to clarify the function of the expanded GPI-reactive cells in AA and PNH patients.

#### Immune Selection of GPI (-) Cells by CD4^+^ Lymphocyte-Mediated Attack in a Murine Model of Graft-Versus-Host Disease

Murakami et al. used a mouse model of PNH to evaluate the ability of GPI (-) cells to outcompete GPI (+) cells in the setting of allogeneic graft-versus-host (GVH) response (41). The investigators specifically focused on CD4^+^ T cell mediated immunity because of previous reports linking HLA class II-restricted autoimmunity to AA—e.g., putative autoreactive CD4^+^ T cell clones were obtained by culturing AA patients’ T cells with autologous hematopoietic progenitors (103, 104), and multiple studies reported an association of HLA DR15 with AA (105, 106). Thus, Murakami et al. used an established model of lethal pancytopenia due to allogeneic incompatibility in MHC class II I-A^b^ gene (bm12) to test the sensitivity of GPI (-) cells to CD4^+^ T cell-mediated immune attack. A mixture of GPI (+) and GPI (-) murine fetal liver cells were transplanted alone or together with bm12-mismatched CD4^+^ T lymphocytes into lethally irradiated B6 control mice. In the presence of co-transplanted bm12 CD4^+^ T cells, the percentages of fetal liver-derived cells at 5 and 8 weeks post-transplant comprised only ~20%, consistent with T cell mediated killing of donor cells. However, GPI (-) cells made up most of the surviving donor cells, indicating that GPI (+) HSPCs were selectively killed, while GPI (-) cells were selectively spared by T cell mediated attack. As CD4^+^ bm12 T cells were gradually lost post-transplant, the fraction of GPI (+) cells increased. At 21 weeks post-transplant, the GPI (-) cell proportions in animals co-transplanted with CD4^+^ bm12 cells were the same as mice that did not receive bm12 CD4^+^ cells.

Murakami et al.’s results reinforced that PNH clonal expansion is not cell-intrinsic and occurs due to external selective pressure in the marrow environment (in this case, CD4^+^ T cell-mediated allogeneic immune attack). Reversion to the original proportions of GPI (-) cells following the disappearance of alloreactive T cells implies that the CD4^+^ T cells likely attacked multipotent progenitors but not the long-term hematopoietic stem cells (HSCs). The immune attack likely spared the long-term HSCs because of their lack of MHC-II expression. Once allogeneic T cells were gone, GPI (+) HSC were able to reconstitute hematopoiesis. While underlying reasons for the competitive advantage of PNH cells in the bm12 allogeneic T cell model were not explicitly studied, one likely contributing factor was the differential presence of CD48 on GPI (-) and GPI (+) cells. CD48 is a GPI-anchored costimulatory molecule on antigen-presenting cells, which contributes to the organization of the immune synapse by binding to CD2 on T cells (107). Pretreatment of mice with monoclonal antibodies against CD2 and CD48 significantly reduced the severity of CD4^+^ (but not CD8^+^) T lymphocyte-induced graft versus host disease (GVHD) and marrow aplasia, supporting a key role of CD48 costimulation in the induction of bone marrow failure by the bm12 allogeneic T cells (108). However, the extent to which the same mechanism contributes to PNH clonal expansion in humans is less certain. Unlike CD48 in mice, the high-affinity ligand for human CD2 is CD58 (LFA-3), which has some homology to CD48 but exists in both GPI-anchored and transmembrane forms (107, 109). Thus, CD48 is unlikely to play the same role in the immune selection of GPI (+) and GPI (-) cells in AA and PNH patients.

## Discussion

The Greek philosopher Aristotle famously remarked on discovering the unknown: “The more you know, the more you realize you don’t know”. This certainly holds true for PNH, which is a fascinating case study of the complex interplay between cell-intrinsic changes due to *PIGA* loss and the external factors that together produce PNH clonal expansion in patients. Landmark studies identified several potential mechanisms that could promote PNH clonal expansion—whether due to the absence of GPI-anchored NKG2D ligands, a missing GPI antigen, loss of costimulatory molecules, or altered cytokine sensitivity. However, the key obstacle to identifying mechanisms relevant to PNH clonal expansion in patients has been an incomplete understanding of the nature of autoimmunity in AA and the connection between PNH and AA.

With recent advances in complement therapeutics (110–114) (115), have we reached the point where mechanistic understanding of PNH origins is no longer irrelevant? Certainly, many PNH patients can readily receive effective therapy to prevent complement-mediated intravascular hemolysis in as few as six infusions per year with current C5 inhibitor therapy (110, 111). Additionally, advances in proximal complement inhibition (115), including oral inhibitors of Factors B and D in late-stage clinical trials (115–119), hold great promise for more effective and convenient treatment of both intravascular and extravascular hemolysis. Yet, despite tremendous advancements, PNH continues to be a significant burden, requiring life-long, very costly immunosuppressive medications, which are not readily available to PNH patients in resource-poor countries. Furthermore, the close connection between PNH and AA suggests that uncovering the mechanisms of PNH emergence will improve our understanding of autoimmunity in AA. An improved understanding of AA and PNH holds promise for the future development of potentially curative therapies and means of prevention for these life-long relapsing and remitting blood diseases.

The recent discovery of MHC class I restriction of immune attack in AA (100, 101) presents new opportunities to revisit clonal expansion of PNH. As such, the ability of PNH cells to escape autoreactive CD8^+^ T lymphocytes should be investigated. PNH cells may evade MHC class I-restricted immune attack due to a deficiency of GPI-anchored costimulatory molecules, similar to the role of CD48 deficiency in promoting GPI (-) cell survival in bm12 CD4*^+^* T cell-mediated mouse model of marrow aplasia (41). Alternatively, PNH cells may have a reduced expression of AA autoantigen (s) or other alterations in immune recognition. Indeed, given the absence of numerous GPI-APs with diverse functions in PNH cells and the more insidious alterations in intracellular protein trafficking and membrane structure, it is likely that several non-mutually-exclusive mechanisms contribute to immune escape and context-dependent enhancements of GPI (-) cell survival.

Although GPI (-) cell expansions in classical PNH and in marrow failure are believed to arise for similar reasons, mechanisms that lead to large expansions of PNH cells may differ from those that result in the more common smaller PNH clones in AA. Differences in clone size may be partly explained by the varying self-renewal potential of cells that incur the initial *PIGA* mutations. Random genetic drift, particularly when occurring in a hypocellular marrow with oligoclonal hematopoiesis, can also lead to stochastic expansions of *PIGA*-mutant cells (14, 41). However, the lack of PNH association with inherited bone marrow failure diseases or with aging, both of which are associated with oligoclonal hematopoiesis and the development of somatic mutations (9, 90), strongly argues that PNH clones, regardless of size, are etiologically related to AA. Proliferative mutations in several oncogenes (e.g., *HMGA2*, *JAK2*, and *BCR-ABL*) have been reported to cause rare cases of PNH disease (120–123). However, these are exceptional cases, while most PNH patients do not carry oncogenic mutations and most non-*PIGA*, non-*HLA* mutations in AA and PNH patients mirror age-related clonal hematopoiesis (e.g., *DNMT3A, TET2, ASXL1, BCOR/BCORL1*) (120, 124–127).

In sum, PNH is a unique example of a clonal blood disease whose development is fostered by AA’s autoimmune bone marrow environment. Recent advancements in disease models and immunologic technologies and the growing understanding of immunologic and marrow failure processes in AA offer new opportunities to crack the Dameshek Riddle (12) and unravel the mechanisms of clonal expansion of PNH cells and autoimmunity in AA.

## Author Contributions

MC drafted the first version of the manuscript. MC, SK, BM, and DB performed critical literature review, wrote and revised the manuscript, and created figures and tables. All authors approved the final manuscript.

## Funding

This work was supported by NIH K08 HL132101 and the Institute for Translational Medicine and Therapeutics of the Perelman School of Medicine at the University of Pennsylvania grant funded by NCATS UL1TR001878 to DB.

## Conflict of Interest

The authors declare that the research was conducted in the absence of any commercial or financial relationships that could be construed as a potential conflict of interest.

## Publisher’s Note

All claims expressed in this article are solely those of the authors and do not necessarily represent those of their affiliated organizations, or those of the publisher, the editors and the reviewers. Any product that may be evaluated in this article, or claim that may be made by its manufacturer, is not guaranteed or endorsed by the publisher.
